# Identifying risk factors involved in the common versus specific liabilities to substance use: A genetically informed approach

**DOI:** 10.1111/adb.12944

**Published:** 2020-07-23

**Authors:** Eleonora Iob, Tabea Schoeler, Charlotte M. Cecil, Esther Walton, Andrew McQuillin, Jean‐Baptiste Pingault

**Affiliations:** ^1^ Department of Behavioral Science and Health University College London London UK; ^2^ Department of Clinical, Educational and Health Psychology, Division of Psychology and Language Sciences University College London London UK; ^3^ Department of Child and Adolescent Psychiatry Erasmus University Medical Center Rotterdam The Netherlands; ^4^ Department of Epidemiology Erasmus University Medical Center Rotterdam The Netherlands; ^5^ MRC Integrative Epidemiology Unit, Bristol Medical School, Population Health Sciences University of Bristol Bristol UK; ^6^ Department of Psychology University of Bath Bath UK; ^7^ Division of Psychiatry University College London London UK; ^8^ Social, Genetic and Developmental Psychiatry Centre King's College London London UK

**Keywords:** common liability, mental health, personality, polygenic risk, substance use

## Abstract

Individuals most often use several rather than one substance among alcohol, cigarettes or cannabis. This widespread co‐occurring use of multiple substances is thought to stem from a common liability that is partly genetic in origin. Genetic risk may indirectly contribute to a common liability to substance use through genetically influenced mental health vulnerabilities and individual traits. To test this possibility, we used polygenic scores indexing mental health and individual traits and examined their association with the common versus specific liabilities to substance use.

We used data from the Avon Longitudinal Study of Parents and Children (*N =* 4218) and applied trait‐state‐occasion models to delineate the common and substance‐specific factors based on four classes of substances (alcohol, cigarettes, cannabis and other illicit substances) assessed over time (ages 17, 20 and 22). We generated 18 polygenic scores indexing genetically influenced mental health vulnerabilities and individual traits. In multivariable regression, we then tested the independent contribution of selected polygenic scores to the common and substance‐specific factors.

Our results implicated several genetically influenced traits and vulnerabilities in the common liability to substance use, most notably risk taking (*b*_standardised_ = 0.14; 95% confidence interval [CI] [0.10, 0.17]), followed by extraversion (*b*_standardised_ = −0.10; 95% CI [−0.13, −0.06]), and schizophrenia risk (*b*_standardised_ = 0.06; 95% CI [0.02, 0.09]). Educational attainment (EA) and body mass index (BMI) had opposite effects on substance‐specific liabilities such as cigarette use (*b*_standardised‐EA_ = −0.15; 95% CI [−0.19, −0.12]; *b*_standardised‐BMI_ = 0.05; 95% CI [0.02, 0.09]) and alcohol use (*b*_standardised‐EA_ = 0.07; 95% CI [0.03, 0.11]; *b*_standardised‐BMI_ = −0.06; 95% CI [−0.10, −0.02]). These findings point towards largely distinct sets of genetic influences on the common versus specific liabilities.

## Graphical abstract

A number of genetically influenced traits and vulnerabilities were associated with the common liability to substance use, most notably risk taking, followed by extraversion and schizophrenia risk. Educational attainment and body mass index had opposite effects on substance‐specific liabilities such as cigarette use and alcohol use. These findings point towards largely distinct sets of genetic influences on the common versus specific liabilities to substance use.

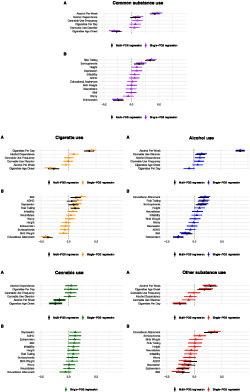

## 1 INTRODUCTION

Substance use is a leading contributor to the global disease and disability burden^1^ and is associated with high societal and economic costs. Of particular public health concern is the problematic use of multiple substances, such as the co‐occurring use of cigarettes, alcohol and cannabis. This pattern of co‐occurrence has pervasive long‐term health implications.^2^ During adolescence and emerging adulthood, the initiation of use of multiple classes of substances may be especially harmful, as it increases the risk of developing the clinical manifestation of a substance use disorder.^3^ To inform prevention strategies, it is therefore essential to understand the origins of such problematic pattern of substance use.

According to the common liability model, the observed correlations between the use of different substances^2^, ^4^, ^5^ can be explained by the presence of a common, nonspecific liability underlying the risk of use of different classes of substances.^6^, ^7^ Support for this model comes from several lines of research. For example, in observational studies, the use of different classes of substances is typically associated with a range of shared individual factors such as mental health vulnerabilities (e.g., schizophrenia, attention deficit and hyperactivity disorder [ADHD]),^8^, ^9^ personality traits (e.g., risk taking),^10^, ^11^ cognitive factors (e.g., educational attainment),^12^ and physical characteristics (e.g., body mass index [BMI]).^13^ Results from twin^4^, ^14^ and genomic studies^15^, ^16^ further indicate that the correlation between the use of different substances stems from a common liability that is largely genetic in nature.

Evidence regarding the common liability model from genome‐wide association studies (GWAS) is more challenging to interpret. So far, GWAS studies have most reliably identified single nucleotide polymorphisms (SNPs) that are associated with the use of particular classes of substances.^16^, ^17^ For example, a replicated finding is the association between the alcohol metabolism gene alcohol dehydrogenase 1B (ADH1B) and alcohol use^16^, ^18^ or the association between the nicotinic receptor gene *CHRNA5* (cholinergic receptor nicotinic alpha 5 subunit) and cigarette use.^16^ While this evidence appears to implicate only substance‐specific genetic effects, recent powerful GWAS studies also identified SNPs with effects shared across two classes of substances (e.g., smoking and alcohol) and identified SNPs that extend beyond ADH1B and CHRNA5.^16^ This highlights the importance of systematically modelling factors that reflect common versus substance‐specific liabilities when assessing genetic influences on substance use.

Genome‐wide findings also implicate that different substance use phenotypes share some polygenic liability with a number of individual traits and vulnerabilities, such as risk taking,^16^, ^19^, ^20^ ADHD,^16^, ^20^, ^21^, ^22^ depression,^21^, ^22^, ^23^ neuroticism,^21^ cognition^20^, ^22^ or schizophrenia.^20^, ^21^, ^22^, ^24^, ^25^ This body of research suggests that the genetic architecture of the common liability may consist of highly polygenic and small indirect effects via a range of genetically influenced mental health vulnerabilities and individual traits. As such, if those traits and vulnerabilities are causally involved in the aetiology of the common liability to substance use, their respective genetic proxies (e.g., genetic variants associated with risk taking) must be associated with the common liability.

In this study, we propose to exploit the polygenic score (PGS) approach to further interrogate the aetiology of the common and substance‐specific liabilities to substance use. A PGS is a continuous index of an individual's genetic risk for a particular phenotype, based on GWAS results for the corresponding phenotype.^26^ PGSs can be used as genetic proxies indexing vulnerabilities and traits to study their role in the common and specific liabilities to substance use. Employing PGSs as proxies for potential risk factors can be conceived as a first step in a series of genetically informed designs to strengthen causal evidence in observational studies.^27^ For example, studies have used PGSs indexing a particular vulnerability or trait, such as depression or psychotic disorders, to test their association with the use of specific classes of substances including cannabis,^28^ alcohol,^29^, ^30^ nicotine^29^, ^30^ or illicit substances.^29^ However, this evidence does not provide insights regarding the aetiology of common versus substance‐specific liabilities. One study has employed the PGS approach to study the effect of a few selected PGSs indexing mental health disorders on the use of multiple substances.^31^ However, important traits and vulnerabilities previously implicated in the aetiology of substance use, including personality traits, cognitive measures and physical characteristics, remain to date untested.

We aimed to triangulate and extend previous phenotypic evidence by integrating genomic data with phenotypic modelling of the common versus specific liabilities to substance use in a longitudinal population‐based cohort. We first generated 18 PGSs, indexing a range of genetically influenced mental health vulnerabilities and traits previously implicated in the aetiology of substance use. Second, we applied the PGS approach to test the association of the 18 genetically influenced vulnerabilities and traits with (a) a common liability to substance use capturing the co‐occurrence of use of alcohol, cigarettes, cannabis and other illicit substances and (b) substance‐specific liabilities that are independent of the common liability. By applying genetically informed methods such as the PGS approach to study refined phenotypes, this investigation has the potential to yield important insights for the aetiology of substance use and inform prevention and treatment programmes.

## 2 METHODS AND MATERIALS

### 2.1 Sample

We analysed data from the Avon Longitudinal Study of Parents and Children (ALSPAC).^32^ Details about the study design, methods of data collection, and variables can be found on the study website (http://www.bristol.ac.uk/alspac/). We used phenotypic data on substance use collected when the study participants were 17, 20 and 22 years of age. Genotype data were available for 7288 unrelated children of European ancestry after quality control (cf. Supporting information for details). Participants were included if they had at least one available substance use measure across the three time points, resulting in a final sample of 4218 individuals. Table S1 presents sample differences between included and nonincluded individuals. Several sample characteristics differed between included individuals and nonincluded individuals, but differences were small in magnitude (observed range *r* = 0.01–0.22). Ethical approval for the study was obtained from the ALSPAC Ethics and Law Committee and the Local Research Ethics Committees.

### 2.2 Measures

#### 2.2.1 Substance use

Substance use (i.e., cigarette, alcohol, cannabis and other illicit substances) was measured at ages 17, 20 and 22. Severity of use of cigarettes, alcohol and cannabis was assessed using validated self‐report questionnaires, namely, the Fagerstrom Test for Nicotine Dependence,^33^ the Alcohol Use Disorders Identification Test^34^ and the Cannabis Abuse Screening Test.^35^ For each scale, total scores were calculated by adding up their item scores (cf. Supporting information for details). For the use of other illicit substances, we computed the total number of illicit substances used in the previous 12 months at each of the three time points (cf. Supporting information for details).

#### 2.2.2 Summary statistics datasets

We collected summary statistics from 32 publicly available GWAS derived from discovery cohorts, which did not include ALSPAC participants (Table S2), indexing domains such as mental health vulnerabilities (e.g., depression), personality (e.g., risk taking), cognition (e.g., educational attainment), physical measures (e.g., BMI) and substance use (i.e., nicotine, alcohol and cannabis use). We chose GWAS indexing either substance use behaviours or individual traits and vulnerabilities that could be plausibly linked to substance use (cf. Section 1). From the initial 32 GWAS, we only included those with a sufficiently large sample (*N* > 20 000 participants) and we excluded several GWAS to avoid content overlap, resulting in a final selection of 18 GWAS summary statistics (cf. Table S3 for further details). References for all GWAS studies used in the analysis and their characteristics can be found in the Supporting information (Tables S2–S3).

### 2.3 Statistical analyses

#### 2.3.1 PGS analysis

Eighteen PGSs were generated utilising PRSice software version 2.2 (http://www.prsice.info/),^26^ based on ALSPAC genotype data and the selected GWAS summary statistics. The PGSs for each individual were calculated as the sum of alleles associated with the phenotype of interest (e.g., schizophrenia), weighted by their effect sizes found in the corresponding GWAS. Clumping was performed in order to remove SNPs in linkage disequilibrium (*r* ^2^ > 0.10 within a 250‐bp window). The PGSs were generated using a single *p*‐value threshold of 1 in order to limit multiple testing while maximising the potential predictive ability of the PGSs.^36^

#### 2.3.2 Trait‐state‐occasion models of substance use

**FIGURE 1:**
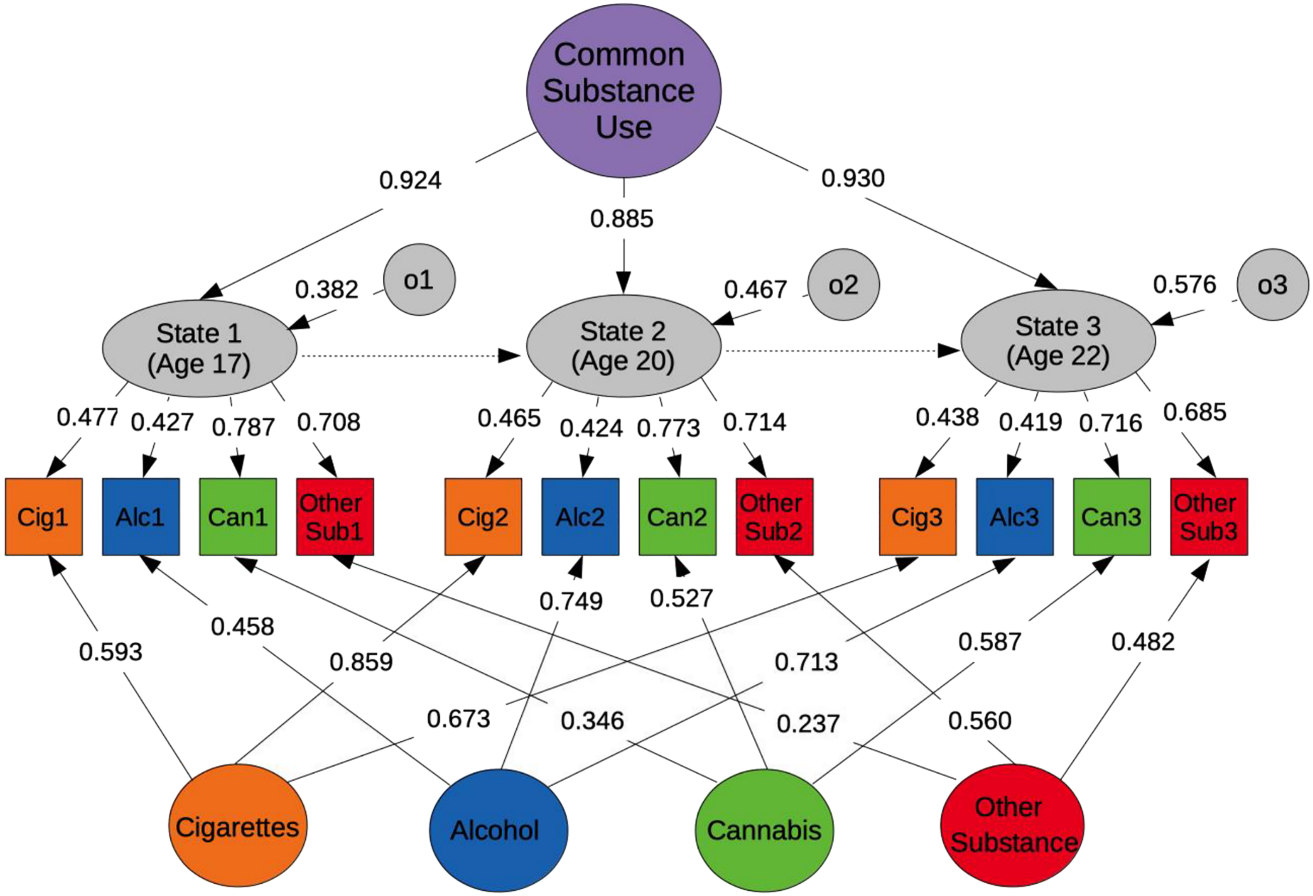
The trait‐state‐occasion model of the common and specific liabilities to substance use. Note. The simplified figure presents the observed measures of substance use (squares) and the latent factors (circles and elliptical shapes). The factors at the bottom represent substance‐specific latent factors. Variances of the latent factors are not shown in the figure and were fixed to 1. Residual variances of the observed variables (not represented) were freely estimated. The estimates reported in the figure represent the standardised factor loadings of the model. o1, occasion factor time 1; o2, occasion factor time 2; o3, occasion factor time 3

All analyses were conducted in R version 3.5.1 using the ‘Lavaan’ package.^37^ First, trait‐state‐occasion (TSO) structural equation models were fitted using the scores for cigarette, alcohol, cannabis and other illicit substance use at each time point.^38^ This approach enabled us to model latent factors of substance use that are stable over time, including (a) a common factor of all substances and (b) substance‐specific factors. Such advanced phenotypic modelling retains a higher degree of precision and specificity compared with simple observed substance use phenotypes. Missing data on the substance use indicators were handled using full maximum likelihood estimation. The model parameters were estimated using robust standard errors due to nonnormality of the substance use scores. The TSO model was tested using available model specifications.^39^ Further details are provided in the Supporting information and in Figure 1. Second, we tested the associations of each PGS with both the common and substance‐specific latent factors (single‐PGS TSO models) in order to explore their individual effects. False discovery rate (FDR) corrected *p* values^40^ are provided to account for multiple testing. Finally, we tested two sets of multivariable TSO models (multi‐PGSs TSO models) for each latent factor, in which we included only those PGSs that remained significant after FDR correction. In the first set, we included PGSs indexing substance use phenotypes (i.e., PGSs indexing dependency and frequency of cigarette, cannabis and alcohol use). In the second set, we included PGSs indexing mental health vulnerabilities and traits. The aim of this multivariable approach was to assess the independent effect of each PGS, controlling for potential pleiotropic effects (i.e., association of a single PGS with an outcome explained by its genetic overlap with other PGSs). All PGS‐regression models were included directly within the TSO models. An example of the Lavaan syntax used for the single and multi‐PGSs models can be found in the Supporting information. All regression models were controlled for sex and population stratification by including 10 principal components as covariates. All PGSs were standardised.

## 3 RESULTS

**FIGURE 2:**
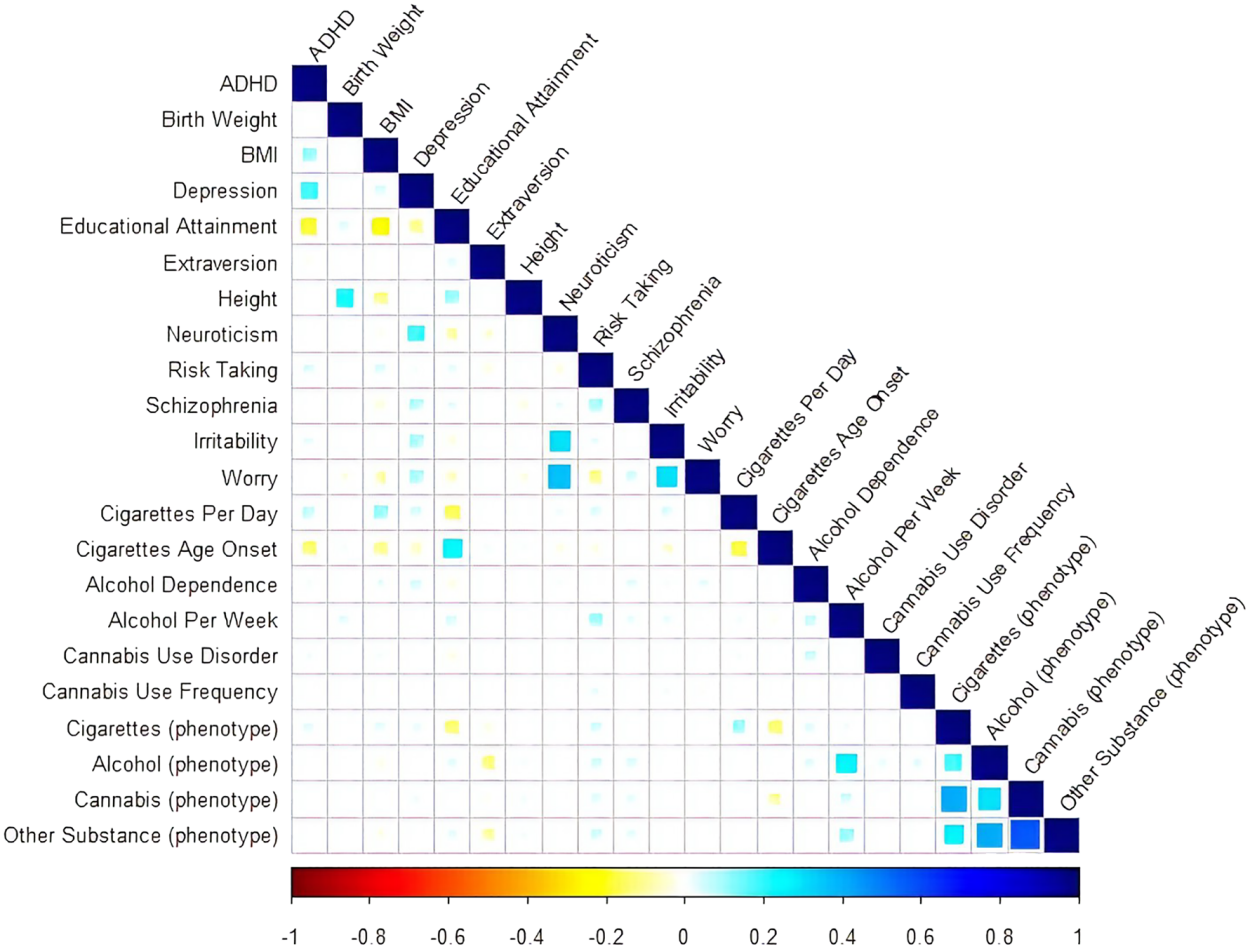
Correlations between the polygenic scores and the phenotype measures assessing substance use (cigarettes, alcohol, cannabis and other illicit substances). Note. ADHD, attention deficit hyperactivity disorder; BMI, body mass index. Blank cells represent nonsignificant coefficients (*p* > 0.05). The correlation estimates and *p* values are reported in Table S5. Included are 18 polygenic scores (Rows 1–18) and 4 phenotype measures assessing substance use (cigarettes, alcohol, cannabis and other illicit substances) across ages 17, 20 and 22 (Rows 19–22)

The descriptive statistics of substance use in our sample can be found in Table S4. Correlations between the 18 PGSs and phenotypic measures of substance use are displayed in Figure 2 and provided in Table S5. The TSO model of substance use fits the data well (*χ* ^2^ (42) = 284.67, *p* < 0.001, Comparative Fit Index (CFI) = 0.952, Root Mean Square Error of Approximation (RMSEA) = 0.037, Standardized Root‐Mean‐Square Residual (SRMR) = 0.058). On average, the common factor accounted for 22% of the total variance in the substance use scores. The substance‐specific factors explained 34% of the variance. Remaining occasion‐specific and residual variances are shown in Table S6.

### 3.1 Effects of the PGSs reflecting substance use

**FIGURE 3:**
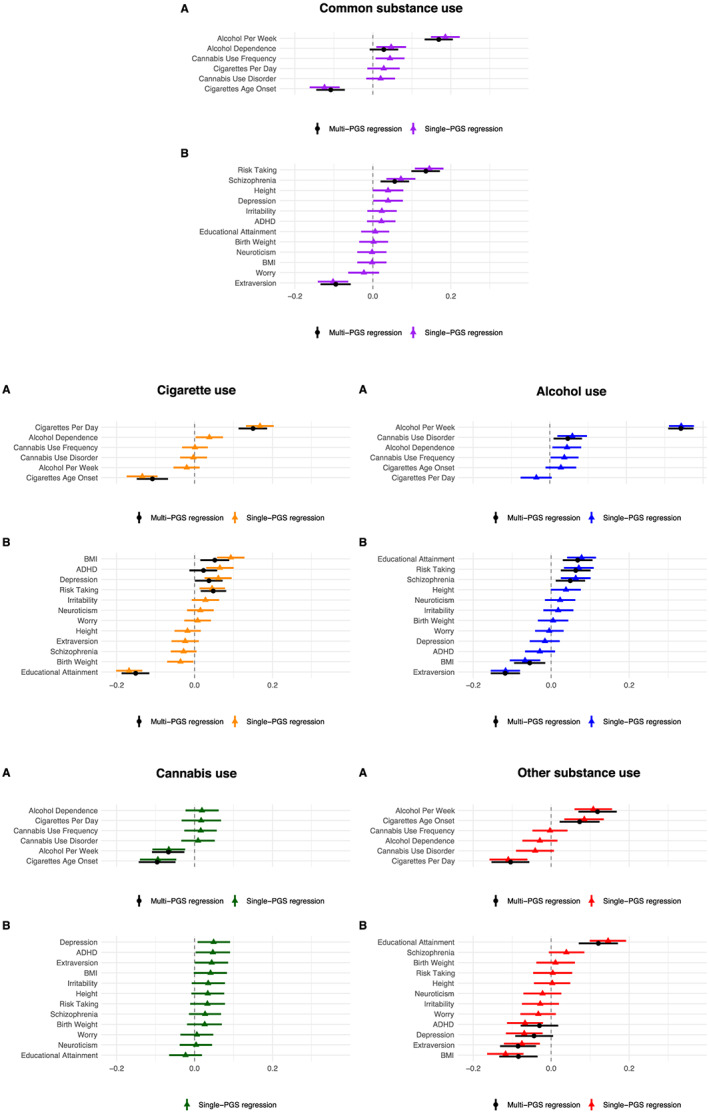
Single‐PGS and multi‐PGSs trait‐state‐occasion models for the common and substance‐specific factors. Note. The estimates represent the standardised regression coefficients and confidence intervals of the single‐ and multi‐PGSs TSO models. ADHD, attention deficit hyperactivity disorder; BMI, body mass index; PGS, polygenic score; TSO, trait‐state‐occasion. Model A: PGSs indexing substance use phenotypes. Model B: PGSs indexing individual vulnerabilities and traits. The explained variance can be obtained by taking the square of the coefficients of the PGSs because both the PGSs and the factors are standardised to a mean of 0 and a variance of 1

The standardised regression coefficients and confidence intervals of the associations of the PGSs with the common and substance‐specific factors are shown in Figure 3 (cf. Tables S7 and S8). As expected, the factors capturing cigarette and alcohol use were predicted by their respective PGSs (e.g., frequency of cigarette/alcohol use), reflecting specific genetic effects (e.g., linked to substance‐specific metabolism). The common factor was independently predicted by two substance use PGSs (age of onset of cigarette use and alcohol frequency), in line with evidence implicating age of onset of cigarette use as a liability marker for initiation of use of other substances.^41^ Other substance‐specific factors were not predicted by their respective PGSs (e.g., cannabis use factor). This could reflect the fact that the GWAS used to derive those PGSs are only of limited power and have not yet succeeded in identifying genetic variants that are substance‐specific in their biological function (e.g., metabolism).^42^

### 3.2 Effects of the PGSs reflecting vulnerabilities and protective traits

#### 3.2.1 Common factor of substance use

In the single‐PGS TSO models, three PGSs (risk taking, extraversion and schizophrenia) were associated with the common factor of substance use after FDR correction and when included in the multi‐PGSs TSO model (Tables S7 and S8, Figure 3). In the multi‐PGSs model, the PGS for risk taking exerted the largest independent effect (*b*_standardised_ = 0.136, *p*_FDR_ < 0.001), followed by the PGS indexing extraversion (*b*_standardised_ = −0.095, *p*_FDR_ < 0.001) and schizophrenia (*b*_standardised_ = 0.056, *p*_FDR_ = 0.003).

#### 3.2.2 Substance‐specific factor: Cigarette use

In the single‐PGS TSO models, five PGSs were associated with the cigarette use factor following FDR correction (educational attainment, BMI, ADHD, depression and risk taking). In the multi‐PGSs TSO model, three PGSs remained associated with the cigarette use factor, including educational attainment (*b*_standardised_ = −0.151, *p*_FDR_ < 0.001) with the largest effect, followed by BMI (*b*_standardised_ = 0.052, *p*_FDR_ = 0.007) and risk taking (*b*_standardised_ = 0.048, *p*_FDR_ = 0.006).

#### 3.2.3 Substance‐specific factor: Alcohol use

In the single‐PGS TSO models, five PGSs were associated with the alcohol use factor (extraversion, educational attainment, risk taking, BMI and schizophrenia), all of which remained significant following FDR correction and in the multi‐PGSs TSO model. The largest effect was found for extraversion (*b*_standardised_ = −0.118, *p*_FDR_ < 0.001), followed by educational attainment (*b*_standardised_ = 0.068, *p*_FDR_ < 0.001), risk taking (*b*_standardised_ = 0.063, *p*_FDR_ = 0.002), BMI (*b*_standardised_ = −0.055, *p*_FDR_ = 0.009) and schizophrenia (*b*_standardised_ = 0.049, *p*_FDR_ = 0.014).

#### 3.2.4 Substance‐specific factor: Cannabis use

None of the PGSs was associated with the cannabis use factor.

#### 3.2.5 Substance‐specific factor: Other illicit substance use

In the single‐PGS TSO models, five PGSs were associated with the factor representing other illicit substance use following FDR correction (educational attainment, BMI, extraversion, depression and ADHD). In the multi‐PGSs TSO model, three PGSs remained independently associated, including educational attainment (*b*_standardised_ = 0.121, *p*_FDR_ < 0.001), extraversion (*b*_standardised_ = −0.085, *p*_FDR_ < 0.001) and BMI (*b*_standardised_ = −0.084, *p*_FDR_ = 0.002).

## 4 DISCUSSION

This study is the first genomic investigation using the PGS approach to examine the contribution of a range of individual traits and vulnerabilities to both common and specific liabilities to substance use. We highlight two important findings. First, our results implicate a number of genetically influenced mental health vulnerabilities and personality traits in the common liability to substance use, namely, PGSs indexing high risk taking, low extraversion and schizophrenia liability. Second, we identified a distinct set of risk factors that independently contributed to substance‐specific liabilities, such as PGSs indexing educational attainment and BMI. In the following section, we will discuss (a) insights for the aetiology of substance use, (b) findings regarding the common liability, (c) findings regarding the substance‐specific liabilities, (d) implications for the prevention and treatment of substance use and (e) limitations.

### 4.1 Insights for the aetiology of substance use

In this study, we exploited the PGS approach as a genetically informed method^43^ to strengthen inference on risk and protective factors involved in liabilities to substance use, thereby enabling triangulation of previous phenotypic evidence with distinct sources of bias (e.g., traditional observational evidence). Using the PGS approach, our results helped to tease apart some of the genetic predispositions (e.g., PGS indexing schizophrenia liability) that indirectly contribute to common and substance‐specific liabilities to substance use. In particular, different sets of genetically influenced mental health vulnerabilities and traits are likely to be involved in common versus substance‐specific liabilities. Importantly, all associations found in this study can be conceptualised as indirect effects of genetically influenced traits and vulnerabilities. To illustrate, our findings suggest that a genetic liability to risk taking could lead to greater risk‐taking behaviour, which in turn could affect an individual's propensity to engage in substance use irrespective of the class of the substance. However, it should be noted that the PGS approach relies on a number of key assumptions (see Section 4.5). As such, we cannot rule out the possibility that confounders impact on the associations between PGSs and our substance use outcomes.

### 4.2 Risk and protective factors involved in the common liability to substance use

Our results confirm previous findings of a common liability that partly underlies the use of different classes of addictive substances, such as cigarettes, alcohol, cannabis and other illicit substances.^6^, ^44^ Regarding its origins, our findings reveal that a genetic liability to high risk taking, low extraversion and schizophrenia contributes to the common liability to substance use. This corroborates previous phenotypic evidence, which reported associations between substance use and similar traits and vulnerabilities.^8^, ^10^, ^11^, ^45^ Intriguingly, a genetic predisposition for risk taking was most robustly associated with a common liability to substance use, but only to a lesser extent with substance‐specific liabilities (cf. next paragraph). This indicates that individuals susceptible to risk taking are more likely to use an array of different substances, irrespective of their class. Similarly, a genetic predisposition to extraversion was most strongly associated with the common liability to substance use, whereas its associations with substance‐specific liabilities were weaker. Thus, high extraversion may protect against the use of various substances. Furthermore, the common liability was influenced by genetic risk for schizophrenia. Taken together, these findings are in line with the notion that the use of various substances could partly reflect a self‐medication strategy for those individuals more vulnerable to psychopathology and maladaptive personality traits.^46^ This is in line with theories implicating the reward system as a common pathway underlying the use of multiple substances—a system altered in distressed individuals and for whom the use of substances may represent a mean to restore homeostasis.^47^ Finally, our results suggest that shared genetic effects among different substances of use are substantially polygenic in nature, involving many genetic variants exerting indirect and small effects (e.g., polygenic association via risk taking). Future large GWAS may therefore benefit from modelling a common liability to substance use, similar to recent genome‐wide attempts aiming to identify common genetic variation underlying psychiatric traits.^48^, ^49^

### 4.3 Risk and protective factors involved in substance‐specific liabilities

Our results also showed that a substantial proportion of the phenotypic variation in substance use could not be explained by a common liability. Using the PGS approach to identify genetically influenced risk and protective factors involved in the substance‐specific liabilities revealed three patterns of associations. First, (a) we identified a set of factors that were linked to both the common liability to substance use, as well as to substance‐specific liabilities. Second, (b) several factors were linked to substance‐specific liabilities but did not contribute to the common liability. Third, (c) some traits previously implicated in substance use were not associated with any of the substance‐specific liabilities.

Regarding (a), we found that all factors involved in the common liability including a genetic predisposition for risk taking, extraversion and schizophrenia also contributed to the liability to alcohol use. Hence, the aetiologies of these two liabilities (i.e., alcohol vs. common) are partly based on overlapping risk factors. At the same time (b), our results showed that two individual traits—BMI and educational attainment—were not linked to the common liability but predicted substance‐specific liabilities. Interesting results emerged regarding the direction of the identified associations. For example, we found that a predisposition for high educational attainment increased the risk of alcohol and illicit substance use but reduced the risk of cigarette use. This is consistent with the notion that education makes people less likely to smoke cigarettes^50^ due to an increased knowledge of its adverse health consequences. At the same time, greater education may provide more opportunities to consume alcohol and access other substances, as indicated by previous observational evidence.^51^ Opposite effects were also present for BMI. Here, a genetic predisposition for high BMI increased the risk of cigarette use, while reducing the risk of alcohol and other illicit substance use. The same pattern of associations has been reported in observational studies. For example, compared with normal weight adolescents, obese adolescents were at reduced risk of alcohol and illicit substance use, but had an elevated risk of cigarette use.^13^ As nicotine is known to suppress appetite, this may suggest that adolescents with a greater predisposition to high BMI could smoke more in an attempt to control their appetite.^52^

Finally (c), some of the previously implicated risk factors (e.g., neuroticism and ADHD)^9^, ^10^ were not associated with the common or substance‐specific liabilities in our sample. First, this could reflect a lack of power of the PGSs used in the analysis. However, we used powerful PGSs (e.g., neuroticism, derived from a GWAS with *N* > 160 000) that have been shown to predict rare outcomes in comparable samples.^53^ Second, some PGSs were associated with substance use liabilities only in less controlled models (e.g., ADHD and depression predicting other illicit substance use only in single‐PGS but not multi‐PGSs models). In addition to power issues, this may indicate that the effects of ADHD/depression were explained by potentially co‐occurring traits that we included in our multivariable models.

### 4.4 Implications for the prevention and treatment of substance use

Our findings offer insights into the aetiology of substance use and have relevant implications for the prevention and treatment of substance use. First, we identified a set of individual vulnerabilities and traits, namely, risk taking, extraversion and schizophrenia, which contributed to the general liability to substance use. Hence, prevention and treatment programmes aiming to reduce substance use across substances in adolescents may benefit from focusing on those vulnerabilities and traits. For example, there is promising evidence from randomised controlled trials showing reductions in substance use following interventions targeting abilities related to risk taking (e.g., self‐regulation) in adolescents.^54^ Our results also highlight that it is important to target those individuals at greatest risk of developing a problematic pattern of substance use based on pre‐existing vulnerabilities such as schizophrenia. Hence, in adolescents with prodromal symptoms, particular emphasis may need to be placed on the prevention of substance use. Finally, it is important to better understand the mechanisms underlying some of the substance‐specific associations found in this study (e.g., high BMI as a risk factor for cigarette use) in order to design more effective prevention and intervention strategies.

### 4.5 Limitations

By using genetic proxies that are more robust to confounding,^27^ the PGS approach retains key advantages over simple phenotypic associations. However, as with any inference method, the PGS approach relies on a number of assumptions not directly testable (e.g., horizontal pleiotropy and reverse causation). For example, dynastic effects mean that the observed association between the child's PGS and substance use outcomes may actually reflect environmentally mediated genetic effects originating in the parents, rather than genetic effects originating in the child. In this instance, the child PGS is not an adequate proxy of the child vulnerability or trait. Employing the PGS approach in within‐family genetic designs can deal with several of these limitations including dynastic effects^55^ and should be considered in future. In addition, sensitivity analyses as part of Mendelian randomisation methods are available and can help to assess potential violations (e.g., certain forms of pleiotropy). Such analyses will be possible once GWAS summary statistics for our outcomes of interest (i.e., common and specific liabilities to substance use) are available. Because our measures represent substance use behaviours, the findings cannot be generalised to specific substance use disorders. It could be possible that the genetics of substance use is shared across substances, whereas the genetics of substance use disorders might be substance‐specific and related to their specific pharmacology. Follow‐up investigations integrating other related liabilities are therefore essential to further inform aetiological questions. These may include, for instance, liabilities reflecting different facets of complex substance use phenotypes (e.g., common liability to substance abuse or dependence), different patterns of use (e.g., common liability to age of onset of substance use and frequency of substance use), different classifications of substances of use (e.g., abuse of stimulants vs. depressants) or liabilities reflecting addictive behaviours more generally (e.g., gambling). It should also be noted that, unlike for alcohol, cigarette and cannabis use, a validated clinical screening instrument was not available in this sample for other illicit substances. This needs to be considered when interpreting findings for this measure. Finally, this study focused on a sample of young adults. Future research should therefore expand to other age groups to assess if the contribution of some of the identified factors (e.g., risk taking) to substance use is adolescent‐delimited.

## 5 CONCLUSION

Our findings reveal that distinct sets of genetically influenced vulnerabilities and protective factors are likely to be involved in the common versus substance‐specific liabilities to substance use. In particular, a genetic predisposition to high risk taking, low extraversion and schizophrenia may be associated with the individual's susceptibility to the use of any type of substance. Additionally, genetic predispositions related to educational attainment and BMI were related to the use of multiple specific substances, although in opposite directions. Prevention programmes in adolescents may benefit from focusing on these vulnerabilities and protective factors.

## AUTHOR CONTRIBUTIONS

Iob, Schoeler and Pingault had full access to all of the data in the study and take responsibility for the integrity of the data and the accuracy of the statistical analyses.

*Study concept and design*: Pingault, Iob and Schoeler. Acquisition, analysis, or interpretation of data: All authors. Drafting of the manuscript: Pingault, Iob and Schoeler. *Critical revision of the manuscript for important intellectual content*: All authors. *Statistical analysis*: Iob, Schoeler and Pingault. *Obtained funding*: Pingault. *Administrative, technical, or material support*: Pingault, Iob and Schoeler. *Study supervision*: Pingault.

## FUNDING INFORMATION

This research is funded by grant MQ16IP16 from MQ: Transforming Mental Health (Dr Pingault). The UK Medical Research Council and Wellcome (Grant ref: 102215/2/13/2) and the University of Bristol provide core support for ALSPAC. GWAS data were generated by Sample Logistics and Genotyping Facilities at Wellcome Sanger Institute and LabCorp (Laboratory Corporation of America) using support from 23andMe. A comprehensive list of grants funding is available on the ALSPAC website (http://www.bristol.ac.uk/alspac/external/documents/grant‐acknowledgements.pdf). Miss Iob is funded by the ESRC‐BBSRC Soc‐B Centre for Doctoral Training (ES/P000347/1). Dr. Cecil received funding from the European Union's Horizon 2020 research and innovation programme under the Marie Skłodowska‐Curie grant agreement No 707404.

## ROLE OF THE FUNDER/SPONSOR

The funding sources had no role in the design and conduct of the study; collection, management, analysis and interpretation of the data; preparation, review or approval of the manuscript; and decision to submit the manuscript for publication.

## ADDITIONAL CONTRIBUTIONS

We are grateful to all the families who took part in this study, the midwives for their help in recruiting them, and the whole ALSPAC team, which includes interviewers, computer and laboratory technicians, clerical workers, research scientists, volunteers, managers, receptionists and nurses.

## Supporting information

**Table S1.** Early sample characteristics for excluded versus included participants.
Table S2. Overview GWAS summary statistics.
Table S3. Summary of GWAS summary statistics excluded and included in the analysis.
Table S4. Descriptive statistics of the four substance use measures at age 17, 20, and 22.
Table S5. Estimates of the correlations between the 18 PGSs and the mean scores of the measures of substance use (cigarettes, alcohol, cannabis, and other substances) across age 17, 20 and 22.
Table S6. TSO model parameters.
Table S7. Single‐PGS TSO models.
Table S8. Multi‐PGSs TSO models.
