# Supplementary material for: Identifying risk factors involved in the common versus specific liabilities to substance use: A genetically informed approach
Source: Addict Biol. 2020 Jul 23;26(3):e12944. doi: 10.1111/adb.12944 (PMC8427469; doi:10.1111/adb.12944)
Supplement: Supplementary file 1 — Table S1. Early sample characteristics for excluded versus included participants. Table S2. Overview GWAS summary statistics. Table S3. Summary of GWAS summary statistics excluded and included in the analysis. Table S4. Descriptive statistics of the four substance use measures at age 17, 20, and 22. Table S5. Estimates of the correlations between the 18 PGSs and the mean scores of the measures of substance use (cigarettes, alcohol, cannabis, and other substances) across age 17, 20 and 22. Table S6. TSO model parameters. Table S7. Single‐PGS TSO models. Table S8. Multi‐PGSs TSO models. [file ADB-26-e12944-s001.docx]

**Supplementary Information**

**eMETHODS**

**Genotyping and quality control procedures**

Data was drawn from the Avon Longitudinal Study of Parents and Children (ALSPAC)^1^, a birth cohort study that recruited 14,541 pregnant woman with expected dates of delivery between 1^st^ April 1991 and 31^st^ December 1992, resulting in over 14,062 live births. 13,988 children were alive at 1 year of age. A total of 9,912 ALSPAC children were genotyped using the Illumina HumanHap550 quad chip genotyping platforms by 23andme subcontracting the Wellcome Trust Sanger Institute, Cambridge, UK and the Laboratory Corporation of America, Burlington, NC, US. Quality control was carried out in PLINK^2^, adhering to standard guidelines^3^^,4^ and implementing the ALSPAC protocol for genetic data^5^. In brief, individuals were excluded on the basis of gender mismatches; minimal or excessive heterozygosity (+/- 3 SD from the samples' heterozygosity rate mean); disproportionate levels of individual missingness (>3%) and evidence of cryptic relatedness (>10% Identity by Descent, IBD). Population stratification was assessed by multidimensional scaling analysis and compared with Hapmap II (release 22) European descent (CEU), Han Chinese, Japanese and Yoruba reference populations. All individuals with non-European ancestry (including self-reported) were removed. SNPs with a minor allele frequency of < 1%, a call rate of < 95%, a Hardy-Weinberg equilibrium of *p* < 5E-7 were removed. Non-autosomal SNPs were removed. 7,288 subjects and 6,953,294 SNPs passed these quality control filters.

Principal components analysis (PCA) was used to generate the top 15 principal components. PCA was performed on a subset of 4,126,412 SNPs that passed the quality control procedure described above, after pruning to remove markers in linkage disequilibrium (r^2^ > 0.1 within a 50 SNP window) using PLINK (indep-pairwise 250 50 0.1).

**Substance Use Measures**

*Cigarette Use*

Cigarette use was measured using the Fagerstrom Test^6^, a validated measure of physical dependence on cigarette smoking which has been widely used across different countries in both clinical and non-clinical settings. The questionnaire consists of six items assessing the frequency and compulsion of cigarette consumption as well as nicotine dependence. The total Fagerstrom score can range between 0 and 10. The total Fagerstrom score was included as a continuous outcome in our models, indexing severity of cigarette use.

*Alcohol Use*

Alcohol use was ascertained using the Alcohol Use Disorders Identification Test (AUDIT)^7^. This is a 10-item screening tool developed by the World Health Organisation to assess recent alcohol consumption, alcohol dependence symptoms, and alcohol-related problems. The AUDIT has been shown to provide a valid and reliable measure of alcohol use problems across gender, age, and cultures^8^. The total AUDIT score ranges from 0 to 40, with higher scores indicating greater alcohol use problems. The total AUDIT score was included as a continuous outcome.

*Cannabis Use*

Cannabis use was assessed with the Cannabis Abuse Screening Test (CAST)^9^, a validated tool for the screening of cannabis use disorders among adolescents and young adults. The questionnaire consists of six items pertaining to different aspects of problematic cannabis use such as non-recreational use (smoking alone or before midday), memory disorders, unsuccessful attempts to quit, and problems linked to cannabis consumption. The overall CAST score in ALSPAC ranges between 0 and 24 and was included as a continuous outcome.

*Other illicit substances*

No validated questionnaire was available to assess the use of other illicit substances. We therefore created an indicator representing the total number of other illicit substances used in the previous 12 months at each of the three different time points. These included: cocaine, amphetamines, inhalants, sedatives, hallucinogens, and opioids. The total score (1-6) was included as a continuous outcome.

**Analytical Strategy**

*Trait-State-Occasion (TSO) models.* Out of the N = 7288 genotyped participants, we included N=4218 participants with at least one substance use measure across all time points. The TSO models were tested using the specification model provided in Newsom^10^. The total scores of the four substance use measures at each time point were used as observed variables of three state factors, which in turn were indicators of a latent substance use factor. The model included a separate occasion factor for the residual variance remaining after the trait factor variance was accounted for. In addition, four substance-specific (i.e. method) factors were specified accounting for the unique variance in each substance use measure across the three time points (Figure 1, manuscript). The adequacy of the TSO models was evaluated using Root Mean Square Error of Approximation (RMSEA), Comparative Fit Index (CFI), and Standardized Root-Mean-Square Residual (SRMR). RMSEA and SRMR are both measures of absolute fit. Acceptable model fit is indicated by RMSEA/ SRMR values less than .05. CFI is a comparative fit index which should be greater than .90^11^.

**Example of the Lavaan syntax used for the single-PGS and multi-PGSs TSO models of the common liability factor (i.e. ‘eta’).**

**Single-PGS TSO model**

model<- ' #state factors;

eta1 =~ NA*FT_total17.5_s + (lambda1)*FT_total17.5_s + (lambda2)*audit_tot17.5_s + (lambda3)*cast_tot17.5_s + (lambda4)*drug_tot17.5_s

eta2 =~ NA*FT_total20_s + (lambda1)*FT_total20_s + (lambda2)*audit_tot20_s + (lambda3)*cast_tot20_s + (lambda4)*drug_tot20_s

eta3 =~ NA*FT_total22_s + (lambda1)*FT_total22_s + (lambda2)*audit_tot22_s + (lambda3)*cast_tot22_s + (lambda4)*drug_tot22_s

#occasion factors

etao1 =~ 1*eta1

etao2 =~ 1*eta2

etao3 =~ 1*eta3

#trait factor

eta =~ 1*eta1 + 1*eta2 + 1*eta3

eta1 ~~ 0*eta1

eta2 ~~ 0*eta2

eta3 ~~ 0*eta3

#covariances

eta ~~ 0*etao1

#method factors with effects coding;

etam1 =~ NA*FT_total17.5_s + (lambda5)*FT_total17.5_s + (lambda6)*FT_total20_s + (lambda7)*FT_total22_s

etam2 =~ NA*audit_tot17.5_s + (lambda8)*audit_tot17.5_s + (lambda9)*audit_tot20_s + (lambda10)*audit_tot22_s

etam3 =~ NA*cast_tot17.5_s + (lambda11)*cast_tot17.5_s + (lambda12)*cast_tot20_s + (lambda13)*cast_tot22_s

etam4 =~ NA*drug_tot17.5_s + (lambda14)*drug_tot17.5_s + (lambda15)*drug_tot20_s + (lambda16)*drug_tot22_

eta ~~ 0*etam1 + 0*etam2 + 0*etam3 + 0*etam4 +eta

etam1 ~~ 0*etao1 + 0*etao2 + 0*etao3 + 0*etam2 + 0*etam3 + 0*etam4

etam2 ~~ 0*etao1 + 0*etao2 + 0*etao3 + 0*etam3 + 0*etam4

etam3 ~~ 0*etao1 + 0*etao2 + 0*etao3 + 0*etam4

etam4 ~~ 0*etao1 + 0*etao2 + 0*etao3

#complex constraints for effects coding identification;

#Model constraint:

lambda1 == 4 - lambda2 - lambda3 - lambda4

lambda5 == 3 - lambda6 - lambda7

lambda8 == 3 - lambda9 - lambda10

lambda11 == 3 - lambda12 - lambda13

lambda14 == 3 - lambda15 - lambda16

#single-PGS regression

eta ~ PGS + sex + pca1 + pca2 + pca3 + pca4 + pca5 + pca6 + pca7 + pca8 + pca9 + pca10

'

fitmodel<- sem(model, data=alsp3, information = "expected", missing = "ML", estimator = "MLR")

summary(fitmodel, fit.measures=TRUE, standardized=TRUE, rsquare=TRUE, ci = TRUE)

**Multi-PGSs TSO model**

model<- ' #state factors;

eta1 =~ NA*FT_total17.5_s + (lambda1)*FT_total17.5_s + (lambda2)*audit_tot17.5_s + (lambda3)*cast_tot17.5_s + (lambda4)*drug_tot17.5_s

eta2 =~ NA*FT_total20_s + (lambda1)*FT_total20_s + (lambda2)*audit_tot20_s + (lambda3)*cast_tot20_s + (lambda4)*drug_tot20_s

eta3 =~ NA*FT_total22_s + (lambda1)*FT_total22_s + (lambda2)*audit_tot22_s + (lambda3)*cast_tot22_s + (lambda4)*drug_tot22_s

#occasion factors

etao1 =~ 1*eta1

etao2 =~ 1*eta2

etao3 =~ 1*eta3

#trait factor

eta =~ 1*eta1 + 1*eta2 + 1*eta3

eta1 ~~ 0*eta1

eta2 ~~ 0*eta2

eta3 ~~ 0*eta3

#covariances

eta ~~ 0*etao1

#method factors with effects coding;

etam1 =~ NA*FT_total17.5_s + (lambda5)*FT_total17.5_s + (lambda6)*FT_total20_s + (lambda7)*FT_total22_s

etam2 =~ NA*audit_tot17.5_s + (lambda8)*audit_tot17.5_s + (lambda9)*audit_tot20_s + (lambda10)*audit_tot22_s

etam3 =~ NA*cast_tot17.5_s + (lambda11)*cast_tot17.5_s + (lambda12)*cast_tot20_s + (lambda13)*cast_tot22_s

etam4 =~ NA*drug_tot17.5_s + (lambda14)*drug_tot17.5_s + (lambda15)*drug_tot20_s + (lambda16)*drug_tot22_s

eta ~~ 0*etam1 + 0*etam2 + 0*etam3 + 0*etam4 +eta

etam1 ~~ 0*etao1 + 0*etao2 + 0*etao3 + 0*etam2 + 0*etam3 + 0*etam4

etam2 ~~ 0*etao1 + 0*etao2 + 0*etao3 + 0*etam3 + 0*etam4

etam3 ~~ 0*etao1 + 0*etao2 + 0*etao3 + 0*etam4

etam4 ~~ 0*etao1 + 0*etao2 + 0*etao3

#complex constraints for effects coding identification;

#Model constraint:

lambda1 == 4 - lambda2 - lambda3 - lambda4

lambda5 == 3 - lambda6 - lambda7

lambda8 == 3 - lambda9 - lambda10

lambda11 == 3 - lambda12 - lambda13

lambda14 == 3 - lambda15 - lambda16

#multi-PGSs regression

eta ~ PGS1 + PGS2 + PGS3 + PGS4 + PGS5 + sex + pca1 + pca2 + pca3 + pca4 + pca5 + pca6 + pca7 + pca8 + pca9 + pca10

'

fitmodel<- sem(model, data=alsp3, information = "expected", missing = "ML", estimator = "MLR")

summary(fitmodel, fit.measures=TRUE, standardized=TRUE, rsquare=TRUE, ci = TRUE)

| eTable 1. Early sample characteristics for excluded versus included participants. | | | | | | | | | |
| --- | --- | --- | --- | --- | --- | --- | --- | --- | --- |
|  | **Non-included** | | | **Included** | | | **Group comparisons** | | |
|  | *Mean (%)* | *SD* | *N* | *Mean (%)* | *SD* | *N* | *p-value^*^* | *r^*^* | *N* |
| Sex (% female)^a^ | 43.9 |  | 7690 | 56.9 |  | 4218 | <.001 | 0.198 | 11908 |
| Parental divorce (% yes)^a^ | 17.7 |  | 7647 | 11.5 |  | 4192 | <.001 | 0.162 | 11839 |
| Parental mental illness (%yes)^a^ | 4.0 |  | 7647 | 4.8 |  | 4192 | 0.046 | -0.052 | 11839 |
| Social class^b^ | 3.29 | 1.23 | 6227 | 2.9 | 1.14 | 3728 | <.001 | -0.217 | 9955 |
| Birthweight (g) | 3420.98 | 545.63 | 7614 | 3443.55 | 513.55 | 4188 | 0.025 | 0.023 | 11802 |
| Mood score^c^ | 15.97 | 6.03 | 5900 | 16.34 | 5.69 | 3748 | 0.002 | 0.039 | 9648 |
| Social achievement score^d^ | 19.16 | 3.93 | 6200 | 19.21 | 3.74 | 4046 | 0.517 | 0.005 | 10246 |
| Note. ^*^*p-value* estimates from significance tests, including t-tests (continuous variables) or chi-square tests (binary variables). *r* coefficients obtained from Spearman’s Rho correlation tests. ^a^Reported in % for binary variable. ^b^ Based on occupation, higher scores indicating lower social class. ^c^ Completed by mother (age: 6 months).^d^ Completed by mother (age: 18 months). SD = standard deviation. The correlation coefficients presented here index small effects, with small effects corresponding to r=±0.1 and medium effects to r= =±0.3^12^. | | | | | | | | | |

| eTable 2. Overview GWAS summary statistics. | | | | | | |
| --- | --- | --- | --- | --- | --- | --- |
| Trait | **Sample Size** | **Case** | **Control** | **SNP-based Heritability** | **Year published** | **Reference** |
| ADHD | 53,293 | 19099 | 34194 | 0.236 | 2017 | Demontis D, Walters RK, Martin J, et al. Discovery of the first genome-wide significant risk loci for ADHD. BioRxiv. 2017:145581. |
| Agreeableness | 17,375 |  |  | 0.016 | 2012 | de Moor MHM, Costa PT, Terracciano A, et al. Meta-analysis of genome-wide association studies for personality. Mol Psychiatry. 2012;17(3):337-349. doi:10.1038/mp.2010.128. |
| Alcohol Dependence | 38,686 | 10206 | 28480 | 0.096 | 2018 | Walters, R. K., Polimanti, R., Johnson, E. C., McClintick, J. N., Adams, M. J., Adkins, A. E., … Team, 23andMe Research. (2018). Transancestral GWAS of alcohol dependence reveals common genetic underpinnings with psychiatric disorders. Nature Neuroscience, 21(12), 1656–1669. https://doi.org/10.1038/s41593-018-0275-1 |
| Alcohol per week | 941,280 |  |  | 0.041 | 2019 | Liu, M., Jiang, Y., Wedow, R., Li, Y., Brazel, D. M., Chen, F., … Vrieze, S. (2019). Association studies of up to 1.2 million individuals yield new insights into the genetic etiology of tobacco and alcohol use. Nature Genetics. https://doi.org/10.1038/s41588-018-0307-5 |
| Anorexia nervosa | 14,477 | 3495 | 10982 | 0.243 | 2017 | Duncan L, Yilmaz Z, Gaspar H, et al. Significant locus and metabolic genetic correlations revealed in genome-wide association study of anorexia nervosa. Am J Psychiatry. 2017;174(9):850-858. doi:10.1176/appi.ajp.2017.16121402. |
| Anxiety Disorder (factor score) | 18,186 |  |  | 0.070 | 2016 | Otowa T, Hek K, Lee M, et al. Meta-analysis of genome-wide association studies of anxiety disorders. Mol Psychiatry. 2016;21(10):1391-1399. doi:10.1038/mp.2015.197. |
| Autism spectrum disorder | 15,954 | 7387 | 8567 | 0.290 | 2017 | The Autism Spectrum Disorders Working Group of The Psychiatric Genomics Consortium. Meta-analysis of GWAS of over 16,000 individuals with autism spectrum disorder highlights a novel locus at 10q24.32 and a significant overlap with schizophrenia. Mol Autism . 2017;8(1):21. doi:10.1186/s13229-017-0137-9. |
| Bipolar disorder | 16,731 | 9250 | 16731 | 0.364 | 2011 | Psychiatric GWAS Consortium Bipolar Disorder Working Group. Large-scale genome-wide association analysis of bipolar disorder identifies a new susceptibility locus near ODZ4. Nat Genet. 2011;43(10):977-983. doi:10.1038/ng.943. |
| Birth weight | 205,475 |  |  | 0.122 | 2018 | Neale Lab. Uk Biobnak GWAS results - March 2018. http://www.nealelab.is/uk-biobank. Published 2018. |
| Body mass index | 681,275 |  |  | 0.276 | 2018 | Yengo L, Sidorenko J, Kemper KE, et al. Meta-analysis of genome-wide association studies for height and body mass index in~ 700,000 individuals of European ancestry. bioRxiv . 2018:274654. |
| Cannabis Use Disorder  (ordinal measure) | 8,754 |  |  | Not available | 2016 | Sherva R, Wang Q, Kranzler H, et al. Genome-wide Association Study of Cannabis Dependence. JAMA Psychiatry.2016;73(5):472-480. doi: 10.1001/jamapsychiatry.2016.0036. Severity, Novel Risk Variants, and Shared Genetic Risks |
| Cannabis Use Frequency | 24,798 |  |  | 0.040 | 2018 | Neale Lab. Uk Biobnak GWAS results - March 2018. http://www.nealelab.is/uk-biobank. Published 2018. |
| Cigarette use (age of onset) | 341,427 |  |  | 0.047 | 2019 | Liu, M., Jiang, Y., Wedow, R., Li, Y., Brazel, D. M., Chen, F., … Vrieze, S. (2019). Association studies of up to 1.2 million individuals yield new insights into the genetic etiology of tobacco and alcohol use. Nature Genetics. https://doi.org/10.1038/s41588-018-0307-5 |
| Cigarette use (per day) | 337,334 |  |  | 0.080 | 2019 | Liu, M., Jiang, Y., Wedow, R., Li, Y., Brazel, D. M., Chen, F., … Vrieze, S. (2019). Association studies of up to 1.2 million individuals yield new insights into the genetic etiology of tobacco and alcohol use. Nature Genetics. https://doi.org/10.1038/s41588-018-0307-5 |
| Conscientiousness | 17,375 |  |  | 0.073 | 2012 | de Moor MHM, Costa PT, Terracciano A, et al. Meta-analysis of genome-wide association studies for personality. Mol Psychiatry . 2012;17(3):337-349. doi:10.1038/mp.2010.128. |
| Cross disorder | 61,220 | 33332 | 27888 | Not available | 2013 | Cross-Disorder Group of the Psychiatric Genomics Consotium. Identification of risk loci with shared effects on five major psychiatric disorders: a genome-wide analysis. Lancet . 2013;381(9875):1371-1379. doi:10.1016/S0140-6736(12)62129-1. |
| Depressive symptoms | 161,460 |  |  | 0.048 | 2016 | Okbay A, Baselmans BML, De Neve J-E, et al. Genetic variants associated with subjective well-being, depressive symptoms and neuroticism identified through genome-wide analyses. Nat Genet. 2016;48(6):624-633. doi:10.1038/ng.3552. |
| Diagnosis of depression | 173,005 | 59851 | 113154 | 0.077 | 2018 | Wray NR, Ripke S, Mattheisen M, et al. Genome-wide association analyses identify 44 risk variants and refine the genetic architecture of major depression. Nat Genet. 2018;50(5):668-681. doi:10.1038/s41588-018-0090-3. |
| Educational attainment | 766,345 |  |  | 0.106 | 2018 | Lee JJ, Wedow R, Okbay A, et al. Gene discovery and polygenic prediction from a genome-wide association study of educational attainment in 1.1 million individuals. Nat Genet. 2018;50(8):1112-1121. doi:10.1038/s41588-018-0147-3. |
| Extraversion | 17,375 |  |  | 0.041 | 2012 | de Moor MHM, Costa PT, Terracciano A, et al. Meta-analysis of genome-wide association studies for personality. Mol Psychiatry . 2012;17(3):337-349. doi:10.1038/mp.2010.128. |
| Extraversion (Item Response Theory) | 63,030 |  |  | 0.050 | 2015 | van den Berg SM, de Moor MHM, McGue M, et al. Harmonization of Neuroticism and Extraversion phenotypes across inventories and cohorts in the Genetics of Personality Consortium: an application of Item Response Theory. Behav Genet . 2014;44(4):295-313. doi:10.1007/s10519-014-9654-x. |
| Height | 693,529 |  |  | 0.149 | 2018 | Yengo L, Sidorenko J, Kemper KE, et al. Meta-analysis of genome-wide association studies for height and body mass index in~ 700,000 individuals of European ancestry. bioRxiv . 2018:274654. |
| Intelligence | 269,867 |  |  | 0.184 | 2018 | Savage JE, Jansen PR, Stringer S, et al. Genome-wide association meta-analysis in 269,867 individuals identifies new genetic and functional links to intelligence. Nat Genet. 2018;50(7):912-919. doi:10.1038/s41588-018-0152-6. |
| Internalizing symptoms | 4,596 |  |  | Not available | 2014 | Benke KS, Nivard MG, Velders FP, et al. A genome-wide association meta-analysis of preschool internalizing problems. J Am Acad Child Adolesc Psychiatry. 2014;53(6):667-676.e7. doi:10.1016/j.jaac.2013.12.028. |
| Irritability | 345,231 |  |  | 0.118 | 2018 | Neale Lab. Uk Biobnak GWAS results - March 2018. http://www.nealelab.is/uk-biobank. Published 2018. |
| Loneliness  (linear trait) | 10,760 |  |  | 0.160 | 2017 | Gao J, Davis LK, Hart AB, et al. Genome-wide association study of loneliness demonstrates a role for common variation. Neuropsychopharmacology . 2017;42(4):811-821. doi:10.1038/npp.2016.197. |
| Neuroticism | 168,105 |  |  | Not available | 2018 | Turley P, Walters RK, Maghzian O, et al. Multi-trait analysis of genome-wide association summary statistics using MTAG. Nat Genet. 2018;50(2):229-237. doi:10.1038/s41588-017-0009-4. |
| Obsessive compulsive disorder | 9,725 | 2688 | 7037 | 0.324 | 2018 | Arnold PD, Askland KD, Barlassina C, et al. Revealing the complex genetic architecture of obsessive–compulsive disorder using meta-analysis. Mol Psychiatry . 2018;23(5):1181-1188. doi:10.1038/mp.2017.154. |
| Openness | 17,375 |  |  | 0.106 | 2012 | de Moor MHM, Costa PT, Terracciano A, et al. Meta-analysis of genome-wide association studies for personality. Mol Psychiatry . 2012;17(3):337-349. doi:10.1038/mp.2010.128. |
| Risk taking | 436,236 |  |  | 0.053 | 2018 | Clifton EAD, Perry JRB, Imamura F, et al. Genome–wide association study for risk taking propensity indicates shared pathways with body mass index. Commun Biol. 2018;1(1):36. doi:10.1038/s42003-018-0042-6. |
| Schizophrenia | 105,318 | 40675 | 64643 | 0.412 | 2018 | Pardiñas AF, Holmans P, Pocklington AJ, et al. Common schizophrenia alleles are enriched in mutation-intolerant genes and in regions under strong background selection. Nat Genet. 2018;50(3):381-389. doi:10.1038/s41588-018-0059-2. |
| Worry | 348,219 |  |  | 0.089 | 2018 | Nagel M, Jansen PR, Stringer S, et al. Meta-analysis of genome-wide association studies for neuroticism in 449,484 individuals identifies novel genetic loci and pathways. Nat Genet. 2018;50(7):920-927. doi:10.1038/s41588-018-0151-7. |

| eTable 3. Summary of GWAS summary statistics excluded and included in the analysis. | | |
| --- | --- | --- |
| Included GWASs | **Excluded GWASs (Risk Factors)** | **Reasons for**  **exclusion^1^** |
| Risk Factors | Cross Disorder | 1 |
| ADHD | Internalising | 1, 2 |
| Birth weight | Loneliness | 2 |
| Body mass index | OCD | 2 |
| Depression | Anorexia | 2 |
| Educational attainment | Autism | 2 |
| Extraversion (Item Response Theory) | Depression Symptoms | 1 |
| Height | Extraversion | 1, 2 |
| Neuroticism | Intelligence | 1 |
| Risk taking | Agreeableness | 2 |
| Schizophrenia | Anxiety | 2 |
| Irritability | Bipolar Disorder | 2 |
| Worry | Conscientiousness | 2 |
|  | Openness | 2 |
| Substance Use | ^1^Exclusion criteria: 1 = Construct Overlap; 2 = GWAS < 20,000  **Note.**   1. Construct overlap refers to related different PGSs tapping into similar underlying constructs (e.g. intelligence and educational attainment). In such cases, only one of the PGSs was retained. 2. The cut-off of >20,000 was chosen as a conservative threshold, given that previous studies on substance use exploiting GWAS summary statistics applied a threshold of at least 10,000 individuals^13^. 3. It should be noted that there are important differences between the selected GWAS datasets in terms of their sample size, quality of outcome measures, and representativeness of the study participants. However, all of the k=18 GWAS included in this study were of quality that was deemed sufficient to test our models, as indexed by their size (only those with N >20,000 were included) and their level of prior evaluation, either through independent reviewers (k=15 GWAS published in peer reviewed papers) or research committees (k=3 GWAS included from the Biobank). | |
| Alcohol Dependence |  |  |
| Alcohol Per Week |  |  |
| Cigarettes Age Onset |  |  |
| Cigarettes Per Day |  |  |
| Cannabis Use Disorder |  |  |
| Cannabis Use Frequency |  |  |

| **eTable 4. Descriptive statistics of the four substance use measures at age 17, 20, and 22.** | | | | |
| --- | --- | --- | --- | --- |
|  |  | **Age 17 (%)** | **Age 20 (%)** | **Age 22 (%)** |
| **Alcohol** (AUDIT; total score range: 0-40) | *Non-users* | 4.7 | 2.0 | 5.0 |
|  | *Low Problems (0-8)* | 54.5 | 41.8 | 52.0 |
|  | *Moderate Problems (8-15)* | 36.7 | 42.9 | 37.2 |
|  | *High Problems (16-40)* | 4.1 | 13.3 | 5.8 |
|  | *Mean (SD)* | 7 (4.4) | 9.1 (5.4) | 7.3 (4.8) |
|  | *Missing (%)* | 32.7 | 36.3 | 38.1 |
| **Cigarettes** (Fagerstrom; total score range: 0-10) | *Non-users* | 48.5 | 39.6 | 39.5 |
|  | *Occasional user ^a^* | 39.6 | 42.5 | 48.6 |
|  | *Low Problems (0-2)* | 4.8 | 10.0 | 5.6 |
|  | *Moderate Problems (3-5)* | 4.5 | 5.4 | 4.5 |
|  | *High Problems (6-10)* | 2.5 | 2.6 | 1.7 |
|  | *Mean (SD)* | 0.4 (1.4) | 0.5 (1.4) | 0.4 (1.2) |
|  | *Missing (%)* | 30.9 | 32.2 | 37.3 |
| **Cannabis (**CAST; total score range: 0-24**)** | *Non-users* | 92.4 | 84.6 | 88.6 |
|  | *Low Problems (0-8)* | 5.2 | 12.6 | 9.4 |
|  | *Moderate Problems (9-16)* | 2.1 | 2.4 | 1.9 |
|  | *High Problems (17-24)* | 0.4 | 0.4 | 0.2 |
|  | *Mean (SD)* | 0.9 (2.5) | 1.2 (2.6) | 1.1 (2.3) |
|  | *Missing (%)* | 39.7 | 32.4 | 37.7 |
| **Other substances** (total score range: 0-6) | *Non-users* | 85.8 | 80.0 | 72.6 |
|  | *Low Problems (1-2)* | 10.9 | 14.5 | 18.5 |
|  | *Moderate Problems (3-4)* | 2.6 | 4.4 | 7.0 |
|  | *High Problems (5-6)* | 0.7 | 1.2 | 1.9 |
|  | *Mean (SD)* | 0.3 (0.8) | 0.4 (1.0) | 0.6 (1.2) |
|  | *Missing (%)* | 32.1 | 34.9 | 39.9 |
| **Note.** AUDIT = Alcohol Use Disorders Identification Test. CAST = Cannabis Abuse Screening Test. Fagerstrom = Fagerstrom Test of Nicotine Dependence. Other substances = total number of other illicit substances consumed in the past 12 months (i.e. cocaine, ketamine, heroin etc.). Missing = proportion (%) of missing data in each of the four substance use measures at each of the three time points. SD = Standard Deviation.  ^a^ Occasional users = participants smoking less than one cigarette per week. | | | | |

| **eTable 5. Estimates of the correlations between the *18* PGSs and the mean scores of the measures of substance use (cigarettes, alcohol, cannabis, and other substances) across age 17, 20 and 22.** | | | | | | | | | | | | | | | | | | | | | |
| --- | --- | --- | --- | --- | --- | --- | --- | --- | --- | --- | --- | --- | --- | --- | --- | --- | --- | --- | --- | --- | --- |
|  | ADHD | Birth Weight | BMI | Depression | Educational Attainment | Extraversion | Height | Neuroticism | Risk Taking | Schizophrenia | Irritability | Worry | Cigarettes Per Day | Cigarettes Age Onset | Alcohol Dependence | Alcohol Per Week | Cannabis Use Disorder | Cannabis Use Frequency | Cigarettes (phenotype) | Alcohol (phenotype) | Cannabis (phenotype) |
| ADHD |  |  |  |  |  |  |  |  |  |  |  |  |  |  |  |  |  |  |  |  |  |
| Birth Weight | -0.01 |  |  |  |  |  |  |  |  |  |  |  |  |  |  |  |  |  |  |  |  |
| BMI | 0.13*** | 0.01 |  |  |  |  |  |  |  |  |  |  |  |  |  |  |  |  |  |  |  |
| Depression | 0.20*** | -0.01 | 0.07*** |  |  |  |  |  |  |  |  |  |  |  |  |  |  |  |  |  |  |
| Educational Attainment | -0.19*** | 0.06*** | -0.22*** | -0.11*** |  |  |  |  |  |  |  |  |  |  |  |  |  |  |  |  |  |
| Extraversion | -0.04* | 0.02 | -0.01 | -0.03 | 0.06*** |  |  |  |  |  |  |  |  |  |  |  |  |  |  |  |  |
| Height | -0.03 | 0.22*** | -0.11*** | -0.01 | 0.13*** | -0.02 |  |  |  |  |  |  |  |  |  |  |  |  |  |  |  |
| Neuroticism | 0.03 | -0.03 | -0.04* | 0.18*** | -0.08*** | -0.07*** | -0.02 |  |  |  |  |  |  |  |  |  |  |  |  |  |  |
| Risk Taking | 0.07*** | 0.01 | 0.08*** | 0.04* | 0.05** | -0.05** | 0.00 | -0.05** |  |  |  |  |  |  |  |  |  |  |  |  |  |
| Schizophrenia | 0.02 | 0.00 | -0.05** | 0.11*** | 0.04* | -0.02 | -0.05** | 0.04* | 0.11*** |  |  |  |  |  |  |  |  |  |  |  |  |
| Irritability | 0.04* | 0.01 | 0.02 | 0.11*** | -0.05*** | -0.02 | -0.02 | 0.31*** | 0.05** | 0.03 |  |  |  |  |  |  |  |  |  |  |  |
| Worry | 0.00 | -0.04** | -0.10*** | 0.12*** | -0.07*** | 0.02 | -0.05** | 0.37*** | -0.11*** | 0.09*** | 0.30*** |  |  |  |  |  |  |  |  |  |  |
| Cigarettes Per Day | 0.10*** | -0.01 | 0.13*** | 0.07*** | -0.15*** | 0.00 | -0.02 | 0.04** | 0.07*** | 0.01 | 0.06*** | 0.01 |  |  |  |  |  |  |  |  |  |
| Cigarettes Age Onset | -0.13*** | 0.04* | -0.10*** | -0.08*** | 0.25*** | 0.04* | 0.03* | -0.04** | -0.05** | 0.00 | -0.07*** | 0.00 | -0.18*** |  |  |  |  |  |  |  |  |
| Alcohol Dependence | 0.04* | 0.00 | 0.04** | 0.07*** | -0.04** | 0.02 | -0.02 | 0.04* | 0.03 | 0.07*** | 0.04* | 0.06*** | 0.01 | -0.03 |  |  |  |  |  |  |  |
| Alcohol Per Week | -0.01 | 0.05** | -0.03 | 0.02 | 0.07*** | -0.03 | 0.01 | 0.01 | 0.12*** | 0.06*** | 0.05** | -0.03* | 0.03* | -0.04* | 0.08*** |  |  |  |  |  |  |
| Cannabis Use Disorder | 0.03* | 0.03 | 0.03* | 0.03 | -0.04** | -0.02 | 0.02 | 0.00 | 0.01 | -0.01 | 0.01 | -0.01 | 0.03* | -0.02 | 0.08*** | 0.03 |  |  |  |  |  |
| Cannabis Use Frequency | 0.02 | -0.01 | -0.01 | 0.00 | 0.01 | 0.00 | 0.03 | -0.01 | 0.05** | 0.02 | 0.03* | -0.03* | 0.00 | -0.01 | -0.01 | 0.02 | 0.02 |  |  |  |  |
| Cigarettes (phenotype) | 0.05** | -0.02 | 0.06*** | 0.05** | -0.12*** | -0.05** | 0.00 | 0.01 | 0.08*** | 0.01 | 0.03 | 0.00 | 0.13*** | -0.13*** | 0.05** | 0.04* | 0.02 | 0.02 |  |  |  |
| Alcohol (phenotype) | -0.01 | 0.00 | -0.05** | 0.01 | 0.05*** | -0.12*** | 0.04* | 0.02 | 0.08*** | 0.07*** | 0.02 | -0.01 | -0.02 | -0.02 | 0.05** | 0.26*** | 0.05** | 0.04** | 0.20*** |  |  |
| Cannabis (phenotype) | 0.02 | 0.01 | 0.02 | 0.04* | 0.00 | -0.03* | 0.03* | 0.00 | 0.08*** | 0.05*** | 0.02 | -0.01 | 0.02 | -0.09*** | 0.03 | 0.09*** | 0.01 | 0.03 | 0.42*** | 0.30*** |  |
| Other Substance (phenotype) | -0.02 | 0.00 | -0.05** | 0.01 | 0.06*** | -0.08*** | 0.03* | -0.01 | 0.08*** | 0.06*** | 0.00 | -0.02 | -0.02 | -0.03 | 0.02 | 0.13*** | 0.00 | 0.02 | 0.26*** | 0.41*** | 0.57*** |
| **Note. p< .001 ***; p<.01 **; p<.05*.** | | | | | | | | | | | | | | | | | | | | | |

| eTable 6. TSO model parameters. | | | | |
| --- | --- | --- | --- | --- |
|  | **R-squared** | **R-squared decomposition** | | |
|  |  | *SU Trait* | *Occasion-specific* | *Substance -specific* |
| *Age 17* | | | | |
| Cigarettes | 0.576 | 0.132 | 0.085 | 0.357 |
| Alcohol | 0.396 | 0.115 | 0.074 | 0.206 |
| Cannabis | 0.741 | 0.378 | 0.243 | 0.119 |
| Other substances | 0.555 | 0.308 | 0.198 | 0.047 |
| *Age 20* | | | | |
| Cigarettes | 0.954 | 0.115 | 0.094 | 0.744 |
| Alcohol | 0.789 | 0.104 | 0.085 | 0.599 |
| Cannabis | 0.869 | 0.336 | 0.275 | 0.257 |
| Other substances | 0.870 | 0.286 | 0.234 | 0.349 |
| *Age 22* | | | | |
| Cigarettes | 0.645 | 0.113 | 0.074 | 0.456 |
| Alcohol | 0.645 | 0.114 | 0.075 | 0.455 |
| Cannabis | 0.876 | 0.316 | 0.209 | 0.350 |
| Other substances | 0.677 | 0.293 | 0.194 | 0.190 |
| *Average R-squared* (%) | 72% | 22% | 15% | 34% |
| Note. TSO model without risk factors. TSO = Trait-state-occasion. SU = substance use. The residual variance in the observed variables can be derived for each row by computing 1-R-squared. | | | | |

| eTable 7. Single-PGS TSO models. | | | | | | | | | | | | | | | | | | | | | | | | | | | | |
| --- | --- | --- | --- | --- | --- | --- | --- | --- | --- | --- | --- | --- | --- | --- | --- | --- | --- | --- | --- | --- | --- | --- | --- | --- | --- | --- | --- | --- |
|  | **Common Substance Use** | | | | | | | | **Cigarette Use** | | | | **Alcohol Use** | | | | **Cannabis Use** | | | | | | **Other Substance Use** | | | | | |
| PGSs | Coef. | 95% CI | p | | | p (perm/FDR) | | | Coef. | 95% CI | p | p (perm/FDR) | Coef. | 95% CI | p | p (perm/FDR) | Coef. | | 95% CI | p | | p (perm/FDR) | Coef. | | 95% CI | p | | p (perm/FDR) |
| *Substance Use* | | | | | | | | | | | | | | | | | | | | | | | | | | | | |
| Cigarettes Per Day | 0.028 | -0.014; 0.069 | | 0.189 | | | 0.284 | | 0.168 | 0.132; 0.203 | <0.001 | <0.001 | -0.035 | -0.076; 0.005 | 0.087 | 0.154 | | 0.017 | -0.033; 0.068 | 0.496 | 0.572 | | | -0.110 | -0.158; -0.061 | <0.001 | <0.001 | |
| Cigarettes Age Onset | -0.124 | -0.162; -0.085 | | <0.001 | | | <0.001 | | -0.134 | -0.174; -0.095 | <0.001 | <0.001 | 0.029 | -0.011; 0.069 | 0.155 | 0.245 | | -0.094 | -0.14; -0.047 | <0.001 | <0.001 | | | 0.085 | 0.034; 0.135 | 0.001 | 0.003 | |
| Alcohol Dependence | 0.047 | 0.009; 0.085 | | 0.016 | | | 0.040 | | 0.038 | 0.003; 0.073 | 0.032 | 0.064 | 0.045 | 0.007; 0.082 | 0.019 | 0.041 | | 0.019 | -0.023; 0.062 | 0.378 | 0.473 | | | -0.029 | -0.074; 0.016 | 0.201 | 0.287 | |
| Alcohol Per Week | 0.186 | 0.149; 0.223 | | <0.001 | | | <0.001 | | -0.020 | -0.054; 0.013 | 0.229 | 0.312 | 0.343 | 0.311; 0.376 | <0.001 | <0.001 | | -0.066 | -0.108; -0.024 | 0.002 | 0.006 | | | 0.108 | 0.060; 0.156 | <0.001 | <0.001 | |
| Cannabis Use Disorder | 0.020 | -0.017; 0.057 | | 0.283 | | | 0.369 | | -0.003 | -0.037; 0.032 | 0.872 | 0.934 | 0.059 | 0.020; 0.097 | 0.003 | 0.008 | | 0.009 | -0.034; 0.052 | 0.680 | 0.756 | | | -0.041 | -0.090; 0.007 | 0.097 | 0.162 | |
| Cannabis Use Frequency | 0.044 | 0.007; 0.081 | | 0.019 | | | 0.041 | | 0.001 | -0.032; 0.034 | 0.962 | 0.962 | 0.038 | 0.002; 0.075 | 0.041 | 0.077 | | 0.016 | -0.026; 0.057 | 0.463 | 0.556 | | | -0.003 | -0.048; 0.042 | 0.909 | 0.940 | |
| *Mental Health* | | | | | | | | | | | | | | | | | | | | | | | | | | | | |
| ADHD | 0.022 | -0.015; 0.058 | | 0.243 | | | 0.555 | | 0.065 | 0.030; 0.100 | <0.001 | <0.001 | -0.029 | -0.067; 0.010 | 0.141 | 0.390 | | 0.047 | 0.003; 0.091 | 0.036 | 0.154 | | | -0.067 | -0.113; -0.021 | 0.004 | 0.021 | |
| Depression | 0.039 | 0.001; 0.077 | | 0.046 | | | 0.180 | | 0.061 | 0.026; 0.095 | 0.001 | 0.006 | -0.016 | -0.055; 0.022 | 0.407 | 0.763 | | 0.049 | 0.008; 0.091 | 0.020 | 0.095 | | | -0.069 | -0.116; -0.022 | 0.004 | 0.021 | |
| Worry | -0.023 | -0.063; 0.016 | | 0.249 | | | 0.555 | | 0.008 | -0.026; 0.042 | 0.651 | 1.000 | -0.005 | -0.041; 0.032 | 0.810 | 1.000 | | 0.006 | -0.036; 0.048 | 0.780 | 1.000 | | | -0.033 | -0.079; 0.012 | 0.153 | 0.405 | |
| Schizophrenia | 0.072 | 0.035; 0.109 | | <0.001 | | | <0.001 | | -0.028 | -0.061; 0.005 | 0.097 | 0.312 | 0.063 | 0.025; 0.101 | 0.001 | 0.006 | | 0.027 | -0.014; 0.068 | 0.194 | 0.485 | | | 0.039 | -0.006; 0.085 | 0.090 | 0.300 | |
| *Personality* | | | | | | | | | | | | | | | | | | | | | | | | | | | | |
| Extraversion | -0.102 | -0.141; -0.063 | | <0.001 | | | <0.001 | | -0.024 | -0.059; 0.011 | 0.185 | 0.476 | -0.117 | -0.155; -0.080 | <0.001 | <0.001 | | 0.044 | 0.001; 0.086 | 0.044 | 0.180 | | | -0.075 | -0.121; -0.029 | 0.001 | 0.006 | |
| Irritability | 0.023 | -0.014; 0.061 | | 0.219 | | | 0.533 | | 0.028 | -0.007; 0.063 | 0.115 | 0.334 | 0.018 | -0.020; 0.057 | 0.347 | 0.694 | | 0.035 | -0.007; 0.078 | 0.105 | 0.326 | | | -0.028 | -0.075; 0.020 | 0.253 | 0.555 | |
| Neuroticism | -0.002 | -0.04; 0.035 | | 0.903 | | | 1.000 | | 0.015 | -0.019; 0.049 | 0.398 | 0.762 | 0.023 | -0.016; 0.062 | 0.252 | 0.555 | | 0.004 | -0.038; 0.045 | 0.864 | 1.000 | | | -0.022 | -0.071; 0.026 | 0.360 | 0.704 | |
| Risk Taking | 0.145 | 0.108; 0.181 | | <0.001 | | | <0.001 | | 0.045 | 0.013; 0.078 | 0.007 | 0.035 | 0.071 | 0.033; 0.109 | <0.001 | <0.001 | | 0.033 | -0.011; 0.078 | 0.143 | 0.390 | | | 0.004 | -0.046; 0.054 | 0.884 | 1.000 | |
| *Physical Development* | | | | | | | | | | | | | | | | | | | | | | | | | | | | |
| Birth weight | 0.002 | -0.035; 0.039 | | | 0.904 | | | 1.000 | -0.036 | -0.070; -0.003 | 0.032 | 0.144 | 0.005 | -0.034; 0.044 | 0.803 | 1.000 | | 0.026 | -0.019; 0.07 | 0.260 | 0.557 | | | 0.011 | -0.038; 0.061 | 0.658 | 1.000 | |
| BMI | -0.002 | -0.040; 0.035 | | | 0.912 | | | 1.000 | 0.093 | 0.058; 0.128 | <0.001 | <0.001 | -0.067 | -0.106; -0.028 | 0.001 | 0.006 | | 0.041 | -0.002; 0.083 | 0.060 | 0.208 | | | -0.117 | -0.164; -0.071 | <0.001 | <0.001 | |
| Height | 0.039 | <0.001; 0.078 | | | 0.048 | | | 0.180 | -0.018 | -0.051; 0.016 | 0.303 | 0.620 | 0.038 | -0.001; 0.076 | 0.057 | 0.205 | | 0.034 | -0.008; 0.076 | 0.113 | 0.334 | | | 0.003 | -0.044; 0.049 | 0.904 | 1.000 | |
| *Cognition* | | | | | | | | | | | | | | | | | | | | | | | | | | | | |
| Educational Attainment | 0.006 | -0.030; 0.042 | | | 0.737 | | | 1.000 | -0.168 | -0.201; -0.134 | <0.001 | <0.001 | 0.078 | 0.041; 0.115 | <0.001 | <0.001 | | -0.023 | -0.065; 0.019 | 0.291 | 0.609 | | | 0.146 | 0.099; 0.192 | <0.001 | <0.001 | |
| Note. Data source: ALSPAC, N = 4218. Coef. = standardised regression coefficient. CI = confidence interval. p = p-value. p (perm/FDR) = False Discovery Rate (FDR) corrected p-values. Estimator = MLR (maximum likelihood estimation with robust standard errors). TSO = Trait-State-Occasion. PGS = Polygenic Score. ADHD = Attention deficit hyperactivity disorder. BMI = Body Mass Index. | | | | | | | | | | | | | | | | | | | | | | | | | | | | |

| eTable 8. Multi-PGSs TSO models. | | | | | | | | | | | | | | | | | | | | |
| --- | --- | --- | --- | --- | --- | --- | --- | --- | --- | --- | --- | --- | --- | --- | --- | --- | --- | --- | --- | --- |
| Common Substance Use | | | | | **Cigarette Use** | | | | **Alcohol Use** | | | | **Cannabis Use** | | | | **Other Substance Use** | | | |
| PGSs | Coef. | 95% CI | p | p (perm/FDR) | Coef. | 95% CI | p | p (perm/FDR) | Coef. | 95% CI | p | p (perm/FDR) | Coef. | 95% CI | p | p (perm/FDR) | Coef. | 95% CI | p | p (perm/FDR) |
| *Substance Use* | | | | | | | | | | | | | | | | | | | | |
| Cigarettes Per Day |  |  |  |  | 0.150 | 0.113; 0.186 | <0.001 | <0.001 |  |  |  |  |  |  |  |  | -0.104 | -0.153; -0.056 | <0.001 | <0.001 |
| Cigarettes Age Onset | -0.108 | -0.145; -0.072 | <0.001 | <0.001 | -0.108 | -0.148; -0.068 | <0.001 | <0.001 |  |  |  |  | -0.096 | -0.143; -0.049 | <0.001 | <0.001 | 0.073 | 0.022; 0.124 | 0.005 | 0.006 |
| Alcohol Dependence | 0.028 | -0.008; 0.065 | 0.125 | 0.125 |  |  |  |  |  |  |  |  |  |  |  |  |  |  |  |  |
| Alcohol Per Week | 0.169 | 0.133; 0.205 | <0.001 | <0.001 |  |  |  |  | 0.342 | 0.309; 0.375 | <0.001 | <0.001 | -0.067 | -0.109; -0.026 | 0.001 | 0.001 | 0.119 | 0.070; 0.168 | <0.001 | <0.001 |
| Cannabis Use Disorder |  |  |  |  |  |  |  |  | 0.047 | 0.010; 0.084 | 0.013 | 0.014 |  |  |  |  |  |  |  |  |
| *Individual Vulnerability and Protective Factors* | | | | | | | | | | | | | | | | | | | | |
| ADHD |  |  |  |  | 0.023 | -0.013; 0.058 | 0.209 | 0.221 |  |  |  |  |  |  |  |  | -0.030 | -0.078; 0.018 | 0.227 | 0.227 |
| Depression |  |  |  |  | 0.037 | 0.001; 0.072 | 0.042 | 0.050 |  |  |  |  |  |  |  |  | -0.044 | -0.092; 0.005 | 0.078 | 0.088 |
| Schizophrenia | 0.056 | 0.020; 0.093 | 0.002 | 0.004 |  |  |  |  | 0.049 | 0.012; 0.087 | 0.010 | 0.013 |  |  |  |  |  |  |  |  |
| Extraversion | -0.095 | -0.134; -0.057 | <0.001 | <0.001 |  |  |  |  | -0.118 | -0.155; -0.080 | <0.001 | <0.001 |  |  |  |  | -0.085 | -0.131; -0.039 | <0.001 | <0.001 |
| Risk Taking | 0.136 | 0.099; 0.172 | <0.001 | <0.001 | 0.048 | 0.016; 0.081 | 0.004 | 0.007 | 0.063 | 0.025; 0.101 | 0.001 | 0.002 |  |  |  |  |  |  |  |  |
| BMI |  |  |  |  | 0.052 | 0.015; 0.089 | 0.005 | 0.008 | -0.055 | -0.095; -0.015 | 0.007 | 0.010 |  |  |  |  | -0.084 | -0.133; -0.035 | 0.001 | 0.002 |
| Educational Attainment |  |  |  |  | -0.151 | -0.187; -0.116 | <0.001 | <0.001 | 0.068 | 0.030; 0.106 | <0.001 | <0.001 |  |  |  |  | 0.121 | 0.071; 0.171 | <0.001 | <0.001 |
| Note. Data source: ALSPAC, N = 4218. Coef. = standardised regression coefficient. CI = confidence interval. p = p-value. p (perm/FDR) = False Discovery Rate (FDR) corrected p-values. Estimator = MLR (maximum likelihood estimation with robust standard errors). TSO = Trait-State-Occasion. PGS = Polygenic Score. ADHD = Attention deficit hyperactivity disorder. BMI = Body Mass Index. | | | | | | | | | | | | | | | | | | | | |

**References**

1 Fraser A, Macdonald-Wallis C, Tilling K, Boyd A, Golding J, Davey Smith G *et al.* Cohort Profile: The Avon Longitudinal Study of Parents and Children: ALSPAC mothers cohort. *Int J Epidemiol* 2013; **42**: 97–110.

2 Purcell S, Neale B, Todd-Brown K, Thomas L, Ferreira MAR, Bender D *et al.* PLINK: a tool set for whole-genome association and population-based linkage analyses. *Am J Hum Genet* 2007; **81**: 559–575.

3 Anderson CA, Pettersson FH, Clarke GM, Cardon LR, Morris AP, Zondervan KT. Data quality control in genetic case-control association studies. *Nat Protoc* 2010; **5**: 1564–1573.

4 Marees AT, de Kluiver H, Stringer S, Vorspan F, Curis E, Marie-Claire C *et al.* A tutorial on conducting genome-wide association studies: Quality control and statistical analysis. *Int J Methods Psychiatr Res* 2018; **27**: 1–10.

5 University of Bristol. GWAS data generation. Details as of 26/11/2012. 2012.

6 Fagerström KO, Heatherton TF, Kozlowski LT. Nicotine addition and its assessment. *Ear, nose, throat J* 1990; **69**: 763–765.

7 Saunders JB, Aasland OG, Babor TF, de la Fuente JR, Grant M. Development of the Alcohol Use Disorders Identification Test (AUDIT): WHO Collaborative Project on Early Detection of Persons with Harmful Alcohol Consumption. *Addiction* 1993; **88**: 791–804.

8 Lawford BR, Barnes M, Connor JP, Heslop K, Nyst P, Young RM. Alcohol Use Disorders Identification Test (AUDIT) scores are elevated in antipsychotic-induced hyperprolactinaemia. *J Psychopharmacol* 2012; **26**: 324–329.

9 Legleye S, Karila L, Beck F, Reynaud M. Validation of the CAST, a general population Cannabis Abuse Screening Test. *J Subst Use* 2007; **12**: 233–242.

10 Newsom JT. *Longitudinal Structural Equation Modeling*. Taylor & Francis: New York, 2015.

11 Byrne BM. *Structural equation modeling with Mplus: Basic concepts, applications, and programming.* Routledge/Taylor & Francis Group: New York, US, 2012.

12 Field A. *Discovering statistics using SPSS*. SAGE Publications Limited: London, 2013.

13 Watanabe K, Umićević Mirkov M, de Leeuw CA, van den Heuvel MP, Posthuma D. Genetic mapping of cell type specificity for complex traits. *Nat Commun* 2019; **10**: 1–13.
